# Leishmaniasis in Morocco: Epidemiology, Transmission Dynamics, and the Potential of Artificial Intelligence for Disease Management

**DOI:** 10.1155/japr/4929266

**Published:** 2026-04-01

**Authors:** Chaymaa Harkat, Denis Sereno, Abdelmohcine Aimrane, Moulay Abdelmonaim Hidan, Mohamed Yamani, Kholoud Kahime

**Affiliations:** ^1^ Essaouira Higher School of Technology, Interdisciplinary Laboratory for Research in Environment, Management, Energy and Tourism (LIREMET), Cadi Ayyad University (UCA), Marrakech, Morocco; ^2^ UMR177 Intertryp, IRD, CIRAD, University of Montpellier, Montpellier, France, umontpellier.fr; ^3^ GoInsEct: Global Infecliology and Entomology Research Group, Montpellier, France; ^4^ ENS, Interdisciplinary Research Laboratory in Bioresources, Environment, and Materials, Cadi Ayyad University, Marrakech, Morocco, uca.ma; ^5^ Laboratory of Biotechnology and Natural Resources Valorisation, Ibn Zoh University, Agadir, Morocco; ^6^ Laboratory of Natural Resources and Environment, Polydisciplinary Faculty of Taza, Sidi Mohamed Ben Abdellah University, Taza, Morocco, usmba.ac.ma

**Keywords:** artificial intelligence, disease surveillance, epidemiology, leishmaniases, Morocco, vector control

## Abstract

Leishmaniases are neglected tropical diseases caused by *Leishmania* parasites and transmitted by infected phlebotomine sand flies, and they remain a major public health challenge in Morocco. The burden is dominated by cutaneous leishmaniasis (CL) and visceral leishmaniasis (VL), mainly associated with *Leishmania major*, *Leishmania tropica*, and *L. infantum*. Despite long‐standing national control efforts aligned with the Sustainable Development Goals and the national ambition to eliminate leishmaniasis as a public health problem by 2030, transmission persists and continues to expand in some areas. Climate change, urbanization, and socioeconomic inequities are reshaping vector and reservoir distributions and intensifying human exposure, whereas emerging insecticide resistance threatens the sustainability of current vector‐control approaches. In parallel, recent advances in artificial intelligence (AI) offer new opportunities to strengthen surveillance, diagnosis, and targeted interventions, yet their application to leishmaniasis control in Morocco remains limited. This narrative review synthesizes recent evidence on the epidemiology, transmission cycles, vectors, reservoirs, diagnostic approaches, and control strategies of leishmaniasis in Morocco, and critically discusses how AI‐enabled tools, such as predictive risk mapping, automated vector identification, and image‐based clinical decision support, could help address operational gaps. By integrating AI into existing public health frameworks and reinforcing data quality and capacity building, Morocco could improve early detection, optimize resource allocation, and accelerate progress toward the 2030 elimination goal.

## 1. Introduction

Leishmaniases are parasitic diseases transmitted by phlebotomine sandflies, affecting millions of people worldwide and certain animals (anthropozoonoses), especially preponderant in regions characterized by poverty, limited healthcare infrastructure, and environmental challenges [1–3]. The disease presents in three clinical forms: cutaneous leishmaniasis (CL), visceral leishmaniasis (VL), and mucocutaneous leishmaniasis (ML). Classified as a neglected tropical disease (NTD), leishmaniases remain endemic in 98 countries, impacting an estimated 700,000–1 million people annually, with 26,000–65,000 deaths by VL annually [4].

In Morocco, cutaneous CL, caused by *Leishmania major* and *L. tropica*, primarily affects the skin, often resulting in disfiguring lesions and long‐lasting scars [5, 6]. In contrast, VL, primarily caused by *L. infantum*, is the most severe form of the disease and can be fatal without adequate treatment [7]. According to recent epidemiological reports from the Moroccan Ministry of Health [8], CL remains the dominant form, accounting for 97% of cases, with an incidence rate of 5.62 per 100,000 individuals. VL, by comparison, predominantly affects children and comprises 3% of cases, with an incidence rate of 0.91 per 100,000 people [8]. In 2016, the World Health Organization (WHO) classified Morocco as a high‐burden country for both CL and VL, estimating that 14% of the population is at risk for CL and 10% for VL ([9]. Morocco′s diverse geographical and ecological landscape, which includes arid regions and urbanizing areas, plays a significant role in shaping the distribution of sand fly populations and the transmission patterns of leishmaniasis [10]. The incidence of new cases has increased, with CL primarily caused by *L. major* (54%), which relies on rodents as the main reservoir, and *L. tropica* (43%), for which humans serve as the reservoir. VL is mainly attributed to *L. infantum*, with dogs as the primary reservoir and *Phlebotomus longicuspis*, *Ph. perniciosus*, and *Ph. ariasi* as vector hosts [11]. The epidemiological life cycle is primarily anthroponotic (AL) for *L. tropica* and zoonotic (ZL) for both *L. infantum* and *L. major* [12]. Zoonotic cutaneous leishmaniasis (ZCL), caused by *L. major*, is prevalent in the southern regions, with rodents as the reservoir and transmitted by the vector *Ph. papatasi*. AL CL, caused by *L. tropica*, is found in central and northern areas, transmitted by *Ph. sergenti* and *Ph. chabaudi* and maintained by human and canine reservoirs. Additionally, CL caused by *L. infantum* occurs in northern regions, with dogs as the reservoir [11]. Despite ongoing control efforts over the past decades, the complexity of transmission dynamics, exacerbated by socioenvironmental factors, continues to make disease elimination a formidable challenge.

Morocco has established in 1997 national programs aimed at controlling leishmaniasis, significant barriers remain [13] with the objective is to eliminate by 2030 aligning with the health targets of the 2030 sustainable development agenda [8]. Nevertheless, it is worth noting that, like many other vector‐borne diseases, the transmission dynamics and breeding habitats of sandflies have been significantly affected by rising temperatures and altered precipitation patterns, changes that are particularly relevant in Morocco [7, 14, 15]. Moreover, urbanization has increased contact between human populations and vectors and reservoirs, creating new ecological niches. [7, 15]. Additional factors that make achieving this goal more challenging include socioeconomic disparities, such as poverty and limited access to healthcare [6, 16]. Therefore, there is a pressing need for innovative solutions to bridge existing gaps in surveillance, diagnosis, and control strategies.

Artificial intelligence (AI) presents a novel and promising approach to addressing these challenges. Recent advances in AI, particularly in machine learning (ML) and deep learning (DL), have revolutionized various sectors, including healthcare [17, 18]. AI′s potential to analyze large datasets, predict disease outbreaks, and enhance diagnostic tools makes it a valuable asset in the fight against leishmaniasis [19, 20]. However, AI′s application in control, particularly in Morocco, remains underexplored. This review is aimed at exploring the potential of AI to enhance Morocco′s leishmaniasis control strategies by compiling recent epidemiological data and identifying how AI can contribute to improving vector control, diagnosis, and public health interventions.

To frame this narrative review, we conducted a targeted search of PubMed, Web of Science, and Google Scholar for studies on leishmaniases in Morocco (English/French), combining disease terms (cutaneous, visceral, ML; *Leishmania* spp.) with context terms (epidemiology, vectors, reservoirs, diagnosis, treatment, control, and Morocco), and complemented results with reference‐list screening and official reports from the Morrocan Ministry of Health and the WHO. We included peer‐reviewed articles and programmatic reports presenting original Moroccan data or methodologically explicit syntheses on epidemiology, vector/reservoir ecology, diagnostics, clinical management, control strategies, or socioenvironmental drivers; we excluded items without Moroccan relevance, opinion pieces lacking data, nonverifiable gray literature, and very small case reports (less than five cases) unless offering unique insights. Because this is a narrative (nonsystematic) synthesis, we did not apply a formal risk‐of‐bias tool; instead, two authors appraised sources for credibility using criteria adapted from STROBE/JBI/CASP (design and sampling, diagnostic or taxonomic validity, reporting completeness, handling of confounders, and consistency with independent evidence), prioritizing studies with validated methods (e.g., species‐level typing and standardized bioassays) and transparent reporting. Finally, we assess how emerging AI approaches could strengthen Moroccan leishmaniasis surveillance and control, identifying near‐term opportunities, data and capacity gaps, and policy‐relevant priorities.

Morocco‐specific synthesis is timely because leishmaniasis transmission is evolving under the combined pressure of climate variability, rapid urbanization, and shifting vector–reservoir ecologies, whereas national elimination targets require evidence that is contextualized to local bioclimatic zones and health‐system constraints. In this narrative review, we focus on AI‐relevant opportunities in (i) surveillance and predictive risk mapping, (ii) diagnostic support (image‐ and laboratory‐based decision tools), and (iii) vector/reservoir monitoring and intervention targeting. We neither provide a comprehensive immunological or pathogenesis review, nor do we detail clinical management guidelines or therapeutic trials beyond what is necessary to contextualize diagnostic and control challenges in Morocco.

## 2. Epidemiology of Leishmaniases in Morocco: An Overview

Leishmaniases are part of the NTDs group, infections caused by various pathogens that predominantly affect economically developing countries [7]. Their life cycles are categorized as ZL, in which wild or domestic mammals serve as reservoirs, and AL, where humans are the primary reservoirs. The disease has a significant impact on public health and quality of life, contributing to cycles of poverty and illness in vulnerable populations [21]. Leishmaniasis presents in various forms, including cutaneous, mucocutaneous, and visceral. CL often leads to permanent scarring and social stigma, whereas VL is the most severe and can be fatal if untreated [22]. The disease often leads to chronic conditions that are challenging to manage [23]. Additionally, factors such as population variability and climate change significantly influence the spread and intensity of leishmaniases [24].

### 2.1. ZCL

ZCL is primarily caused by *L. major*, prevalent in southern Morocco, particularly in the High Atlas region. ZCL due to *L. major* is sustained through ZL cycles, with rodents serving as the primary reservoirs. Environmental factors such as the arid climate, high temperatures, and low rainfall create favorable conditions for the proliferation of both vectors and reservoirs, increasing transmission risk [25]. The incidence of ZCL fluctuates with climatic conditions and land‐use changes, which influence the habitats of sand fly vectors and rodent reservoirs. Sporadic cases of ZCL due to *L. infantum* have been described [26] and have also been reported in Morocco [27, 28]. ZCL due to *L. infantum* occurs mainly in northern and central Morocco and is maintained through a ZL transmission cycle in which domestic dogs constitute the primary reservoir. Unlike *L. major*, transmission is associated with more humid and periurban or rural environments, where sand fly vectors thrive in close proximity to human and canine populations. Environmental and anthropogenic factors, including urban expansion, changes in animal husbandry practices, and increased human–dog contact, contribute to transmission dynamics. Climatic conditions influencing vector density and canine infection rates play a key role in shaping the spatial and temporal distribution of ZCL due to *L. infantum*, which often overlaps with VL endemicity.

### 2.2. Anthroponotic Cutaneous Leishmaniasis (ACL)

ACL, caused by *L. tropica*, is endemic to the semiarid central and western provinces along the slopes of the Atlas Mountains. Unlike ZCL, ACL primarily involves human‐to‐human transmission, though domestic dogs have occasionally been identified as secondary reservoirs. Transmission patterns are influenced by factors such as urbanization, population density, and climate variability [10].

### 2.3. Zoonotic Visceral Leishmaniasis (ZVL)

VL in Morocco is caused by *L. infantum*. Although domestic dogs are the primary reservoir of *L. infantum*, wild canids, particularly red foxes and golden jackals, may contribute to the persistence of sylvatic transmission cycles and complicate control strategies [1]. The disease has spread from northern Morocco into smaller towns and rural areas, frequently affecting children. VL is a severe illness that can be fatal if untreated, and its transmission is closely linked to proximity to canine populations [25].

## 3. Epidemiological Trends and Shifts

Over the past two decades, Morocco has seen significant changes in the incidence and geographic distribution of leishmaniasis, particularly cutaneous forms.

### 3.1. ZCL Incidence and Trends

The peak incidence of ZCL occurred in 2010, with 6444 reported cases, primarily concentrated in the Errachidia province, which accounted for 54% of the total cases [29]. However, the number of cases declined sharply in subsequent years, with 2219 cases reported in 2011 and 740 cases in 2012. More recently, between 2017 and 2018, the Zagora pr in southern Morocco recorded over 4000 new cases of ZCL, highlighting the ongoing challenge of controlling the disease in certain areas [30].

### 3.2. ACL Incidence and Trends

Between 2004 and 2013, the mean incidence of ACL was recorded at 139 cases per 100,000 people, with the highest incidence in Chichaoua province (97 cases per 100,000). Females and children aged 0–14 years were found to be particularly vulnerable to ACL infections [7]. The disease has continued to expand into new geographical areas, overlapping with regions affected by ZCL.

### 3.3. ZVL Incidence and Trends

The incidence of ZVL has increased markedly in Morocco since the 1990s, particularly in the northern regions. Between 1990 and 2014, the average annual incidence rate of ZVL was 0.4 cases per 100,000 inhabitants [31]. This rising trend highlights the need for sustained control efforts, particularly given the fatal potential of VL if left untreated.

### 3.4. Canine Leishmaniasis (CanL)

CanL, primarily caused by *L. infantum*, poses a significant challenge in Morocco. The disease affects domestic and stray dogs, which serve as important reservoirs for human transmission. The national prevalence rate of CanL is estimated at 17%, with affected dogs being found across various regions of Morocco [32]. Both *L. infantum* and *L. tropica* have been implicated in CanL, with the latter having ZL potential in some cases.

### 3.5. Conclusion

Evidence from neighboring Maghreb countries supports the idea that transmission foci can shift and (re)appear in areas not classically described as major hotspots. For example, Seklaoui et al. [33] reported autochthonous *L. major* infections in Kabylie (Algeria), interpreted as a signal of changing epidemiology. Such regional signals are relevant for Morocco because shared bioclimatic gradients, population mobility, and vector/reservoir ecologies across North Africa may facilitate the emergence or re‐emergence of transmission zones, reinforcing the need for integrated surveillance and risk mapping approaches.

## 4. Principal *Leishmania* Sand Fly Vectors in Morocco

Sand fly diversity in Morocco is considerable, reflecting the country′s wide range of bioclimatic zones, from humid Mediterranean areas to arid and Saharan environments. To date, 23 phlebotomine sand fly species have been reported, belonging to two genera: *Phlebotomus* and *Sergentomyia* [34]. Among these, species of the genus *Phlebotomus* are of primary medical importance, as they are responsible for the transmission of human leishmaniases. In Morocco, *Ph. papatasi* is the principal vector of *L. major* in southern and pre‐Saharan regions, *Ph. sergenti* is the main vector of *L. tropica* in central and northern areas, and species of the subgenus *Larroussius*, notably *Ph. perniciosus*, *Ph. longicuspis*, and *Ph. ariasi*, play a key role in the transmission of *L. infantum*, particularly in northern and central Morocco.

In Morocco, *Ph. papatasi* and *Ph. sergenti* are well‐established vectors for *L. major* and *L. tropica*, respectively, which cause CL. However, VL is caused by *L. infantum*, of which the primary vectors are *Ph. perniciosus*, *Ph. ariasi*, and *Ph. longicuspis* [35, 36]. A retrospective study in Al Haouz Province identified *Ph. perniciosus* as the most abundant *L. infantum* vector, highlighting the need for vigilant surveillance, particularly during the high‐risk transmission period from March to November [37].

In Zagora Province, *Ph. papatasi* was identified as the most abundant sand fly species, indicating a high risk of *L. major* outbreaks [30]. Meanwhile, *Ph. sergenti*, the primary vector of *L. tropica*, has been documented across all Moroccan ecoregions, suggesting a widespread potential for ACL transmission [38]. A systematic review published in 2022 analyzed the spatiotemporal activity of *Ph. sergenti* across North Africa, Southern Europe, and Asia, revealing that its population peaks in July, with the highest densities observed between June and September. Additionally, this species is associated with higher altitudes, and its distribution is likely affected by climate change, which could in turn impact the transmission of leishmaniasis [39].

Environmental factors, such as temperature and humidity, play a crucial role in shaping the vectorial capacity of sand fly populations. For example, low temperature and humidity levels can directly affect the incubation and maturation periods of *Ph. papatasi*, thereby altering its capacity to transmit *L. major* [40]. In the southern High Atlas Mountains, mean annual temperature and humidity levels align closely with the physiological needs of *Ph. papatasi*, making the region highly conducive to its proliferation ([41]). In northwestern Morocco, however, minimum temperatures below 10°C from November to April significantly limit the emergence and abundance of *Ph. ariasi*, *Ph. perniciosus*, and *Ph. longicuspis* [42]. These vectors also display specific ecological preferences: *Ph. ariasi* typically occurs at altitudes between 1000 and 1400 m, *Ph. perniciosus* is most abundant below 1000 m, and *Ph. longicuspis* inhabits dry areas at altitudes between 800 and 1000 m [43]. Ecological niche modeling (ENM) predicts an increased risk of future ACL outbreaks due to the expanded geographic distribution of *L. tropica* and *Ph. sergenti*, driven by climate change. By 2050, their distribution is expected to widen, emphasizing the importance of understanding environmental and bioclimatic factors that influence vector spread to develop effective prevention and control strategies [44].

## 5. Reservoir Hosts Involved in *Leishmania* Transmission in Morocco

In Morocco, the transmission dynamics of *Leishmania*, with the exception of *L. tropica*, which is primarily AL, are largely sustained within ZL ecosystems. These ecosystems involve wild and peridomestic mammals, particularly rodents from the Rodentia order. *L. infantum*, the causative agent of VL, is closely associated across the Mediterranean region with domestic and stray dogs, as well as wild canines such as foxes [45–48]. Based on previous reports and studies [11, 49], the transmission dynamics of *Leishmania* in Morocco can be described by three primary scenarios: (1) wild reservoirs and vectors share the same ecological niche, with human transmission occurring through a separate vector species with higher anthropophilic tendencies; (2) wild reservoirs and vectors coexist in syntopic environments, where human transmission results from occasional contact with the natural habitat of the reservoirs and vectors; and (3) wild reservoirs, vectors, and humans share the same ecological niche, resulting in continuous transmission cycles that involve all three groups.

For *L. major*, the causative agent of ZCL, the transmission cycle primarily involves rodent reservoirs, including the jird (*Meriones shawi*), the fat sand rat (*Psammomys obesus*), and the great gerbil (*Rhombomys opimus*). In Morocco, *M. shawi* is a key reservoir species and has adapted to peridomestic environments, often foraging near waste disposal sites that provide ideal conditions for sand fly proliferation [25]. The burrows of these rodents create favorable microhabitats for sand fly species such as *Ph. papatasi*, which use these burrows as diurnal resting sites, further facilitating transmission [50]. *P. obesus* has been observed in high densities across various regions in North Africa and the Middle East, including Palestine, Jordan, Algeria, Tunisia, Morocco, Libya, and Egypt [39, 51]. The close associations between *P. obesus* and *M. shawi* in southeastern Morocco, northern Algeria, and Tunisia underscore the critical role these rodent species play in sustaining *L. major* transmission cycles. Studies have shown that the population densities of *Ph. papatasi* correlate strongly with those of *P. obesus*, suggesting an elevated risk of *L. major* transmission in southeastern Morocco and neighboring countries such as northern Algeria, central Tunisia, Libya, and Palestine [51].

This complex network of reservoirs and vectors (see Table 1) highlights the need for a nuanced understanding of the ecological interactions driving leishmaniasis transmission in Morocco, as such insights are crucial for informing effective prevention and control strategies.

**Table 1:** Reservoir and vector hosts of *Leishmania* species reported in Morocco.

*Leishmania* species	Clinical form	Transmission cycle	Main reservoir	Proven vector(s)	Key references
*L. infantum*	Visceral leishmaniasis (VL) and sporadic Zoonotic cutaneous leishmaniasis (ZCL)	Zoonotic	Domestic and stray dogs; wild canids (red fox *Vulpes vulpes*) Golden jackal	*Ph. perniciosus*, *Ph. Longicuspis, Ph. ariasi*	[47]; [48]; [45]; [46]
*L. major*	ZCL	Zoonotic	Rodents: *Meriones shawi*, *Psammomys obesus*, *Rhombomys opimus*	*Ph. papatasi*	[25]; [50]; [39, 51]
*L. tropica*	Cutaneous leishmaniasis (CL)	Primarily anthroponotic	Humans (occasionally dogs reported)	*Ph. sergenti*	[41] [49]

## 6. Key Risk Factors Associated With Leishmaniases in Morocco

### 6.1. Climate Change and Environmental Factors

**Figure 1:**
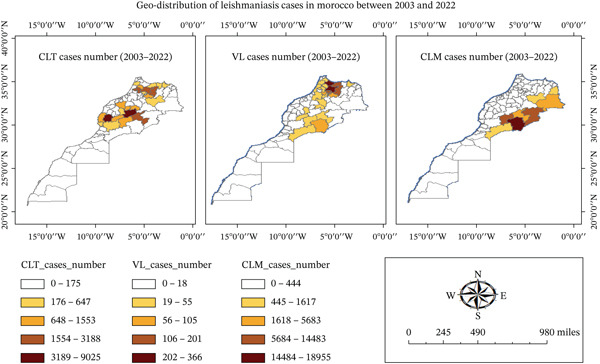
Geographic distribution of human leishmaniases cases in Morocco between 2003 and 2022. CLT, cutaneous leishmaniasis caused by *L. tropica*; VL, visceral leishmaniasis; CLM, cutaneous leishmaniasis caused by *L. major.*

The distribution analysis of human leishmaniases cases from 2003 to 2020 appears to be geographically well‐defined (Figure 1). Environmental changes and human activities often alter natural habitats, leading to the reemergence of leishmaniasis in previously unaffected areas [52]. Epidemiological data indicate that leishmaniasis distribution in Morocco is influenced by climatic conditions such as temperature, rainfall, and humidity [15]. For example, in the southern regions, where temperatures often exceed 35°C, sand fly populations thrive in the arid and semiarid environments conducive to *L. major* transmission [49]. Conversely, in the cooler northern regions, *L. infantum* transmission is more common due to the presence of suitable canine reservoirs and temperate conditions [12, 49].

Climate change has also been linked to shifts in vector distribution, with rising temperatures and altered precipitation patterns impacting sand fly breeding habitats. Bounoua et al. [53] identified a positive association between vegetation indices, precipitation, and ZCL incidence in North Africa, highlighting environmental drivers that remain relevant today. These environmental changes present significant challenges for leishmaniasis control, as they create new breeding grounds for vectors and alter transmission dynamics [53]. The transmission dynamics of VL are closely tied to socioeconomic factors such as poverty and vulnerability rates, along with environmental conditions, including temperature, rainfall, humidity, altitude, and vegetation (assessed via the normalized vegetation index). These factors, coupled with ecological disturbances and environmental shifts, contribute to leishmaniasis remaining a serious public health concern in Morocco. To address this issue effectively, establishing a regional network for information sharing and risk assessment, as well as conducting mass screenings at the regional level, is essential [49].

### 6.2. Socioeconomic Conditions and Urbanization

Socioeconomic factors such as poverty, inadequate housing, and limited access to healthcare are critical determinants of leishmaniasis transmission [6]. Rural areas, particularly those with poor waste disposal systems and close proximity to livestock, are especially vulnerable to the proliferation of sand fly vectors. Urbanization has also altered transmission dynamics, particularly for ACL. Rapid urban growth in provinces like Taounate and Chefchaouen has led to increased contact between human populations and vectors, contributing to the rising incidence of VL in these urbanizing areas [7].

Two regions have been significantly impacted by human visceral leishmaniasis (HVL) due to urbanization. The first region is along a northeast axis, with Taounate province accounting for 16% of cases, and other provinces, including Al Hoceima, Chefchaouen, and Moulay Yacoub, becoming new foci of the disease since 2000 [7]. The second region includes Marrakech‐Tensift‐Al Haouz, Tadla‐Azilal, and Agadir‐Tiznit. From 1997 to 2018, a study conducted in seven northern provinces reported sporadic extensions of VL, with 80% of cases occurring in Al Hoceima. Additionally, CL was documented across 86 sectors, with Larache being the most affected [12].

Molecular typing identified *L. tropica* and *L. infantum* as the predominant species in Taza province, where cases have been increasing since 2010 [54]. The impact of urbanization on leishmaniasis transmission was further highlighted in a retrospective study conducted in the Fez‐Meknes region between 2008 and 2016, which found a positive correlation between urbanization and leishmaniasis incidence. This study emphasized the role of human encroachment in influencing transmission patterns. However, it also noted that poverty rates and urbanization were not always directly correlated with disease incidence, suggesting that other socioenvironmental factors contribute to transmission [55, 56].

A study conducted in Boulemane province reported that the incidence of leishmaniasis increased from six cases in 2000 to 63.65 cases per 100,000 inhabitants in 2009. Researchers suggested that environmental conditions and socioeconomic factors may influence the distribution of *Leishmania* species; however, an ordinary least squares regression (OLSR) analysis indicated that the local microclimate had minimal impact on CL case numbers. The study emphasized the importance of molecular identification for effective control [57]. Additionally, 129 cases of CL were recorded between 2007 and 2010 in Sefrou province, primarily in densely populated, low‐income communities, though no direct link was found between poverty rates and CL cases [58]. In El Hajeb (2013–2017), sociocultural factors were explored, with findings suggesting that seasonal workers from endemic areas contributed to disease spread. Of the 21 new cases reported during this period, 81% were CL.

Overall, the geographical distribution, risk factors, and epidemiological trends of leishmaniasis in Morocco underscore the significant influence of environmental changes, urbanization, and sociocultural factors on the spread of leishmaniasis across the country.

## 7. Advances in the Diagnosis of Leishmaniases in Morocco

Diagnosing leishmaniases accurately is crucial for timely treatment and control of the disease, yet it remains a challenge in many endemic regions, including Morocco, due to the diversity of *Leishmania* species, clinical presentations, and limited resources. The development of diagnostic methods has evolved significantly over the past decades, with both traditional and modern molecular techniques being employed to improve sensitivity and specificity.

### 7.1. Traditional Diagnostic Methods

One of the oldest and still widely used techniques for diagnosing is microscopy, which involves the examination of Giemsa‐stained smears from lesion aspirates, biopsies, or bone marrow samples [59]. This method allows for the direct visualization of *Leishmania* amastigotes within host cells. Despite being considered the gold standard in many settings due to its simplicity and cost‐effectiveness, microscopy has several limitations, among which is the sensitivity of microscopy. This varies depending on the sample quality and the expertise of the practitioner. In the case of CL, it has been reported that sensitivity can range from 30% to 70%, while for VL, it ranges between 50% and 70% [22]. Moreover, microscopy is labor‐intensive and requires significant time to process samples and identify parasites [60]. Nevertheless, although proper training is necessary to achieve proper results, many endemic regions face shortages of trained personnel, particularly in rural settings.

### 7.2. Serological and Immunological Methods

Serological tests, including the indirect fluorescent antibody test (IFAT) and enzyme‐linked immunosorbent assay (ELISA), are widely used for the diagnosis of VL because they detect circulating antibodies against *Leishmania* antigens. One of the most frequently used serological tools for VL is the rK39 rapid diagnostic test (RDT), which identifies antibodies directed against the conserved recombinant rK39 antigen derived from the *L. donovani* complex [61]. However, antibody‐based serological and immunological tests are generally not recommended for the routine diagnosis of CL, as humoral responses in CL patients are often weak, variable, or absent, particularly in localized forms of the disease. In line with this limitation, the systematic review by Gebremeskele et al. [62] reported that RDTs show highly variable performance in CL and are therefore insufficient as standalone diagnostic tools. Consequently, although RDTs offer practical advantages such as speed, simplicity, and minimal infrastructure requirements, their role in CL should remain limited to screening or triage in peripheral settings, and positive or doubtful results should ideally be confirmed by parasitological or molecular methods [62].

The rapid diagnostic tests developed specifically for CL are antigen‐based assays performed on lesion samples rather than blood. These tests detect parasite antigens, such as peroxidoxin, directly in cutaneous lesions and therefore differ fundamentally from rK39 antibody tests. A recent field evaluation conducted in four CL‐endemic provinces in southern Morocco assessed the performance of a CL‐specific antigen RDT and suggested that it may represent an operationally attractive tool in primary healthcare facilities because of its simplicity, rapid turnaround time, and ability to deliver results within a single consultation [6]. Nevertheless, consistent with the systematic review by Gebremeskele et al. [62], the sensitivity of CL RDTs in this setting remained only moderate (65%–79%) and lower than that of RT‐PCR, indicating that false‐negative results may occur and that these tests cannot replace reference parasitological or molecular diagnostic methods. Furthermore, although operationally simple, its cost per strip remains higher than conventional microscopy, and the cost–benefit analysis indicated that a favorable balance was achieved only when the unit price was maintained below 79 MAD (≈USD 7.9–8.1) per test strip [6]. Overall, current evidence supports the use of CL antigen‐based RDTs only as complementary tools for screening or triage of suspected cases in resource‐limited settings, with confirmation by parasitological or molecular techniques whenever feasible.

### 7.3. Molecular Diagnostic Techniques

Polymerase chain reaction (PCR) has revolutionized the diagnosis by offering a highly sensitive and specific method for detecting *Leishmania* DNA. PCR‐based methods are particularly useful for cases with low parasite loads, such as in mucosal leishmaniasis (ML) and chronic forms of CL, where traditional methods often fail [1, 63]. Conventional PCR targets specific regions of *Leishmania* DNA, such as kinetoplast DNA (kDNA) or ribosomal RNA (rRNA) genes, which are conserved among various species. PCR is especially advantageous in its ability to differentiate between species, aiding in species‐specific treatment approaches [1]. Quantitative PCR (qPCR) further enhances the diagnostic potential by not only detecting the presence of DNA but also quantifying the parasite load. This is particularly useful for monitoring the response to treatment and assessing disease progression.

Although PCR methods provide higher sensitivity (up to 100% in some cases), PCR requires sophisticated laboratory infrastructure, trained personnel, and access to reagents, which are not always available in endemic regions, particularly in rural Morocco. Though PCR offers high accuracy, the time required to process samples can delay diagnosis in acute cases. The success of PCR is highly dependent on the quality of the sample. Blood, bone marrow, or tissue samples are commonly used, with varying degrees of success depending on the form of leishmaniasis.

To address these limitations, recent innovations such as loop‐mediated isothermal amplification (LAMP) have been explored as alternatives [63]. LAMP is a molecular diagnostic technique that amplifies DNA under isothermal conditions, offering a faster and cheaper alternative to PCR while maintaining high sensitivity and specificity. LAMP can be used in field settings due to its minimal equipment requirements, making it a promising diagnostic tool for resource‐limited regions like rural Morocco.

Beyond conventional lesion aspirates/biopsies and blood sampling, increasing attention has been given to alternative, minimally invasive or noninvasive specimens to improve feasibility of diagnosis and surveillance in resource‐limited settings. A systematic review and meta‐analysis by Sereno et al. [64] summarized evidence that Trypanosomatidae infections, including leishmaniases, may be detectable in a range of noninvasive or minimally invasive matrices (e.g., body secretions or appendages) using parasitological, serological, antigen‐based, and DNA‐based methods. Although performance varies widely according to the specimen type, parasite burden, and assay platform, this approach is operationally attractive for decentralizing diagnosis and could complement Morocco′s efforts to expand access to testing in peripheral health facilities, particularly when paired with field‐adapted molecular methods such as LAMP.

### 7.4. Novel Diagnostic Approaches

Recent developments in point‐of‐care (POC) diagnostics are transforming the landscape of leishmaniasis diagnosis [65–67]. POC tests are designed to provide immediate results without the need for sophisticated laboratory infrastructure. One such promising development is the use of biosensors and microfluidic devices, which can detect *Leishmania* antigens or nucleic acids at the patient′s bedside. These technologies are still in experimental stages but hold great potential for simplifying the diagnostic process in endemic regions.

### 7.5. Diagnostic Challenges in Morocco

In Morocco, despite global advancements in diagnostic methods, several challenges persist (Table 2).

**Table 2:** Key diagnostic challenges in leishmaniasis detection and management in Morocco.

Diagnostic challenge	Problematic	References
Limited access to advanced diagnostics in rural areas	Rural areas face limited access to advanced diagnostics like PCR, relying instead on microscopy, which can result in misdiagnosis or underdiagnosis	[67]
Sociocultural challenges	The preference for traditional remedies, often delay formal medical diagnosis, highlighting the need for awareness campaigns	[5]; [16]
Socioeconomic challenges	Rapid diagnostic tests for CL (lesion‐based antigen RDTs) may support triage in peripheral facilities, but sustained procurement and cost‐effectiveness remain challenging.	[5]
Genetic diversity of *Leishmania*	The genomic examination of *L. tropica* demonstrates critical genetic variation, confounding analysis and treatment methodologies.	[19]

## 8. Treatment of Leishmaniases in Morocco

Leishmaniasis is still a public health concern in Morocco of which both CL and VL persist as endemic [49, 68]. These are partially characterized by complications that can lead to severe disfigurement in CL or systemic impact in VL cases [49]. The management of effective treatment options of the disease remains critical as well as for the prevention of its long‐term consequences [69]. Yet, Morocco has ensured accessible treatment within its healthcare infrastructure with Glucantime (*Meglumine antimoniate*) as a standard therapeutic agent for all forms across the country [70].

### 8.1. Glucantime as the First‐Line Treatment

Glucantime, a pentavalent antimonial compound, is provided free of charge by the Moroccan Ministry of Health at public health facilities, ensuring that even the most underserved populations have access to necessary care, including a standardized treatment protocol with all required treatment details [70].

The administration of Glucantime is optimized according to the specific form. In the case of CL, it is typically administered intralesionally, directly at the lesion′s margin, thereby allowing for a concentrated treatment of infected tissues [71]. In other words, this method is effective in cases with localized skin lesions. Alternatively, in the cases of VL, the treatment is administered intramuscularly because of the systemic nature of the disease.

Even though Glucantime has shown great treatment effectiveness, it is noteworthy to point out that it has multiple obvious side effects, mainly nausea, vomiting, and potential hepatotoxicity and nephrotoxicity [72]. Consequently, these may deter patients from proceeding throughout the full course of treatment, which may result in incomplete parasite clearance, the risk of relapse, and the possible emergence of drug‐resistant strains [73, 74]. Although Glucantime remains the cornerstone treatment, other factors, beyond its side effects, may impact its effective uptake especially in the Moroccan communities.

### 8.2. Sociocultural Barriers to Treatment

Sociocultural barriers remain a major hindrance to the effective uptake of medical treatments, particularly in rural regions where traditional remedies are often preferred over modern medical options. El‐Mouhdi et al. [32] reported that in underserved areas, communities frequently rely on traditional healers and local remedies, such as herbal treatments or ointments, to manage leishmaniasis. Although these traditional practices may provide temporary relief of symptoms, they are generally ineffective at eliminating the *Leishmania* parasite, as reported by several previous studies ([16]; Mwiti [75]). Although recent research by Et‐Touys et al. [76] has shown promising antileishmanial effects of *Salvia clandestina* (vervain sage), the use of plant products and topical formulations without sufficient knowledge of these practices can lead to more pronounced side effects, complications, or even progression to more severe forms of the disease [16]. For instance, delayed access to conventional treatment of CL may lead to more pronounced consequent lesions and scars on visible body parts, which may have a pronounced side psychological effect especially in young women with facial infestation, often rejected for marriage in Moroccan rural communities [5]. However, misconceptions about the right attitude and prevention methods of leishmaniasis, especially in rural areas such as the provinces in Southeastern Morocco, are probably mean reasons for the preference of folk remedies [5].

Overcoming these sociocultural obstacles calls for specific public health intervention tailored to the specific needs of the population. In southeastern Morocco, a study aimed at reducing CL incidence, which was achieved through an integrated strategy combining community education, vector control, and rodent management. Efforts included promoting hygiene practices to protect against sand fly bites and collaborative initiatives between the Ministry of Health and Ministry of Agriculture (MoA) [77]. The same study highlighted the pertinence of educating communities on the effectiveness of conventional treatment which helped shift their preference to folk medication [77]. Thus, health campaigns must prioritize dispelling myths about the disease and the effectiveness of modern medical interventions, whereas also emphasizing the critical importance of timely diagnosis and treatment with Glucantime as the most effective option.

## 9. Control Strategies

### 9.1. Vector Management Approaches for Targeting Sandfly Populations

Effective sand fly control is central to leishmaniasis prevention and relies on interventions targeting adult and immature vector stages. Integrated vector management strategies include indoor residual spraying (IRS), insecticide‐impregnated bed nets (LLINs), and environmental management to reduce sand fly breeding and resting sites [78].

In Morocco, LLINs have been proposed as a complementary intervention for CL. A household survey conducted in 14 endemic foci reported high ownership rates (94%) but low regular use (34%), reflecting behavioral and cultural barriers that limit effectiveness [79]. A national review similarly identified LLINs as part of integrated vector management but noted the absence of large‐scale Moroccan trials demonstrating a significant impact on transmission [3]. Although WHO‐supported initiatives promote LLINs as sustainable alternatives to DDT (dichloro–diphenyl–trichloroethane), field experience suggests that, in the absence of sustained community adherence, LLINs alone are insufficient, and IRS has shown greater effectiveness in comparable endemic settings [9].

Chemical vector control remains a cornerstone of sand fly management. In Morocco, *Ph. sergenti* and *Ph. papatasi* have been shown to be susceptible to lambdacyhalothrin, DDT, and malathion, although efficacy varies geographically, underscoring the need for continuous entomological surveillance [80]. In areas where IRS was implemented, a marked reduction in leishmaniasis incidence was observed, supporting its effectiveness as a control measure [77].

Insecticide resistance (IR) has been reported sporadically in sand fly populations worldwide, emphasizing the importance of standardized bioassays and molecular monitoring. Prolonged DDT use has led to knockdown resistance (kdr) mutations in *Ph. argentipes* in India, complicating elimination efforts [81], whereas no such mutations were detected in sand flies from Italy, illustrating strong geographic heterogeneity [82]. Field trials across different regions have yielded mixed outcomes, with limited success in India and Venezuela, but substantial reductions in Iran, Greece, and other parts of Latin America (with the exception of Venezuela). For instance, deltamethrin‐based interventions reduced *Ph. perfiliewi* populations by 66% within 24 h in Greece [83].

### 9.2. Reservoir Control and Animal‐Based Interventions

Reservoir management constitutes a complementary pillar of leishmaniasis control, particularly for ZL transmission cycles involving rodents and domestic dogs. In Morocco, control approaches targeting canine reservoirs increasingly emphasize human population management strategies, including Trap–Neuter–Vaccinate–Return (TNVR) programs, alongside vaccination and sterilization of stray dogs. Systematic dog euthanasia is not consistently described as a standard national control strategy, and when applied, it is generally limited to animals that are severely ill or pose a public safety risk in line with animal welfare considerations.

In ZCL caused by *L. major*, rodent reservoir control has been explored as a targeted intervention. Experimental studies have evaluated the use of systemic insecticides such as fipronil administered to rodent reservoirs (*Meriones tristrami* and *Meriones crassus*). These studies demonstrated that sand flies feeding on treated rodents exhibited reduced survival due to sustained levels of fipronil sulfone detected in rodent blood, urine, and feces over 31 days, indicating residual toxicity against sand flies [84].

Such reservoir‐targeted approaches may reduce vector survival indirectly and complement conventional vector control measures. However, their implementation requires careful ecological assessment to avoid unintended environmental consequences and should be integrated into broader, evidence‐based control programs.

### 9.3. Personal Protective Measures, Early Detection, and Treatment of Leishmaniases

In El Hajeb province, Morocco, a study collected 14,590 sandflies from April to December 2019, predominantly identifying *Ph. longicuspis*. The findings highlighted the need for better education and awareness among health professionals and residents regarding sand flies as vectors [85].

A study between 2013 and 2017 in El Hajeb examined the sociocultural aspects of CL, revealing that 43.7% of the population used traditional remedies such as basil, vinegar, and mint. Health education tailored to the sociocultural context is crucial for effective control and prevention [85].

Effective prevention strategies should include hygiene, animal management, and proper irrigation systems [15]. Proper irrigation improves drainage, limits rodent habitats, reduces humidity, lowers human exposure to sandflies, and supports long‐term sustainability in leishmaniasis control. WHO guidelines recommend poisoned bait for controlling reservoirs like *Meriones* species. Morocco′s Ministry of Health also emphasizes rodent control, patient screening, vector control, and environmental sanitation (DELM 2010).

Health education and intersectoral collaboration are essential. The MoA in Morocco implements rodent control and produces poisoned bait for surveillance. Community workers, supervised by the MoA, apply these baits annually for three consecutive years [77].

## 10. AI and Disease Management: A New Frontier for Leishmaniases Control in Morocco

AI can complement existing public health interventions by optimizing resource allocation and improving the effectiveness of vector control programs. For example, AI models could be used to predict the most effective timing and location for IRS and the distribution of insecticide‐treated bed nets. By focusing resources on areas with the highest predicted risk, public health authorities can achieve more significant reductions in disease transmission. AI can also assist in evaluating the impact of interventions. ML algorithms could analyze entomological and epidemiological data to assess how vector control measures influence sand fly populations and leishmaniasis incidence. This information could be used to refine intervention strategies and ensure efficient use of resources.

More broadly, “emergence” in CL is not always a purely biological event; it may also reflect changes in detection capacity, reporting intensity, and the chronic underinvestment typical of NTDs. Sereno [86] argues that discovery and neglect can interact to shape how emergence is perceived, highlighting the value of surveillance systems that reduce diagnostic and reporting blind spots. In Morocco, this perspective supports exploring AI‐enabled approaches (predictive risk mapping, automated vector identification, and image‐based decision support) as tools to strengthen early detection and to better distinguish true ecological expansion from improved case ascertainment.

### 10.1. AI in Understanding and Modeling Infectious Disease Spread

AI has emerged as a powerful tool in understanding the spread of infectious diseases and in predicting future outbreaks. Numerous research projects have focused on utilizing AI to enhance disease modeling, enabling better insights into the mechanisms behind transmission and outbreak patterns. In 2019, Honghao Shi and his team provided key insights into AI′s role in managing the COVID‐19 pandemic. Their research highlighted AI‐based approaches such as compartmental models, agent‐based models, and dynamic parameterization to simulate infectious disease spread more accurately. Traditional models like SEIR (Susceptible‐Exposed‐Infectious‐Recovered), used for infectious disease predictions, were significantly improved through AI‐related techniques during the pandemic [87].

This use of AI to model the spread of diseases can be applied to other infectious diseases like leishmaniasis in Morocco. By integrating environmental, epidemiological, and socioeconomic data, AI can improve outbreak predictions and facilitate more precise interventions, which is especially critical in regions where outbreaks are driven by multiple factors such as climate change and urbanization.

### 10.2. AI for Vector Identification

Vector identification is another area where AI has the potential to revolutionize disease control. DL and computer vision techniques can significantly enhance the accuracy and speed of entomological surveys, particularly for vectors like sandflies, which are responsible for transmitting *Leishmania* parasites. An AI‐based approach, such as Wing Interferential Pattern (WIP) identification, offers an innovative method for near real‐time taxonomic classification of sandflies [88–90].

This is especially relevant for Morocco, where accurate identification of sand fly species is crucial for targeting vector control efforts. AI‐driven tools can rapidly identify species based on morphological characteristics, improving the efficiency of vector surveillance programs and allowing for more targeted interventions to reduce disease transmission.

### 10.3. AI in Clinical Diagnosis

In healthcare, AI technologies such as DL, ML, and big data analytics have transformed diagnostic processes across various medical fields. For example, the MobileNetV2 architecture combined with deep transfer learning has been successfully used to analyze over 33,000 images of skin lesions, achieving diagnostic accuracies as high as 94.42% in classifying skin conditions like basal cell carcinoma (BCC) and squamous cell carcinoma (SCC) [91]. These AI techniques have the potential to be applied to diagnosing CL as well, although research in this specific area remains limited, with only seven studies exploring AI in CL diagnosis.

Talimi et al. [19] have demonstrated that various AI algorithms, including Viola–Jones and YOLOv5, have shown high accuracy in diagnosing CL and other skin conditions. Their study revealed that the DenseNet201 convolutional neural network (CNN) model had the highest diagnostic accuracy for CL. The DenseNet201 model uses high‐resolution images standardized to 224 × 224 pixels, combined with layers like global average pooling and dropout to enhance accuracy and prevent overfitting. The model′s final output, a binary classification, determines the presence or absence of leishmaniasis, making it a valuable tool for early diagnosis and intervention.

Another AI‐based system, using the Viola–Jones object detection algorithm with the Adaboost method, has also demonstrated promising results. This system achieved a sensitivity of 83% and specificity of 35% in detecting *Leishmania*‐infected macrophages and individual parasites, highlighting AI′s potential to improve leishmaniasis diagnosis, particularly in resource‐limited settings [17].

Furthermore, an optimized diagnostic system for three NTDs, including CL, was developed using a dataset of 1054 images, including 262 images of CL. The system achieved a global classification accuracy of 96%, specificity of 94%, and sensitivity of 92%, showcasing its effectiveness in diagnosing early‐stage skin lesions, including leishmaniasis, with faster processing times compared to traditional methods [92].

### 10.4. AI for Leishmaniases Management in Morocco

Despite these promising advances, the use of AI for diagnosing and managing leishmaniasis in Morocco has not yet been fully explored. However, the COVID‐19 pandemic demonstrated the potential of AI in Morocco′s public health sector. Researchers integrated AI through ML and data analytics for environmental monitoring and COVID‐19 management, showing the effectiveness of AI in controlling infectious diseases at a national scale [93].

Ettazi et al. [94] developed and validated several ML models to predict daily COVID‐19 cases and deaths in Morocco, using decision trees, random forests, and support vector machines. These models achieved high accuracy, with an average *R*‐squared value of 0.83 for cases and 0.90 for deaths, underscoring the importance of AI in crisis management. Such models can be adapted for other diseases, including leishmaniasis, to forecast disease incidence and inform resource allocation for prevention and control efforts.

While the potential of AI in leishmaniasis control is considerable, there are challenges to its implementation in Morocco. The lack of high‐quality, annotated datasets for training AI models is one of the most significant obstacles. Data collection on vector populations, disease incidence, and environmental variables must be standardized and integrated into national surveillance systems.

Moreover, limited technical infrastructure and internet connectivity in rural regions pose challenges to deploying AI‐based tools. Investments in infrastructure and capacity‐building initiatives will be necessary to ensure that AI technologies are accessible and scalable across all regions of Morocco. Training healthcare professionals and entomologists in the use of AI tools will also be essential to integrate these technologies into public health practice effectively.

### 10.5. Key Challenges Facing Morocco in Implementing AI for Leishmaniases Management

In conclusion, while AI holds significant potential to enhance leishmaniasis control in Morocco, several challenges must be addressed to enable its effective implementation. The lack of high‐quality, standardized datasets, limited technical infrastructure in rural areas, and insufficient AI expertise hinder the immediate applicability of these technologies. Financial constraints, sociocultural barriers, and ethical considerations related to data privacy further complicate AI adoption in public health. Addressing these challenges through targeted investments in infrastructure, capacity building, and community engagement will be crucial to fully leverage AI′s capabilities in Morocco′s fight against leishmaniasis.

## 11. Conclusion: A Holistic Approach to Leishmaniases Control in Morocco

This review highlights the complex epidemiology of leishmaniasis in Morocco, driven by the diverse interplay between multiple vector species, reservoir hosts, and varied socioenvironmental factors across the country. Morocco′s vast geographical area, which spans multiple bioclimatic zones, from arid deserts to temperate coastal regions, further complicates the disease′s transmission dynamics. These diverse ecological zones support different sand fly species, including *Ph. papatasi*, *Ph. sergenti*, *Ph. perniciosus*, and *Ph. ariasi*, all of which play significant roles in transmitting various *Leishmania* species. This geographic and ecological diversity underscores the need for region‐specific strategies to effectively manage leishmaniasis transmission. IR among sand fly populations is a growing challenge that demands continuous monitoring and a robust response. Additionally, the widespread reliance on traditional remedies points to gaps in public health education, particularly in rural and underserved areas, highlighting the urgent need for targeted awareness campaigns that promote the use of modern healthcare services. Future efforts to combat leishmaniasis in Morocco must prioritize a comprehensive, multifaceted approach. Key strategies should include enhanced vector control, optimized insecticide use, and widespread public health education, particularly in regions most vulnerable to outbreaks. Importantly, these efforts must be supported by continuous research, policy development, and intersectoral collaboration among health, agricultural, and environmental sectors.

Integrating AI into public health initiatives presents a promising avenue for strengthening leishmaniasis surveillance and control. AI has the potential to improve the accuracy of disease modeling, vector identification, and diagnostics, particularly in a country like Morocco, where diverse ecological conditions influence transmission patterns. AI technologies can support predictive modeling based on environmental factors, enabling authorities to preempt outbreaks in high‐risk areas and allocate resources more efficiently.

To effectively address leishmaniasis, several critical actions are needed:

1. **Proactive screening:** Implement regular and widespread screening programs in endemic and high‐risk areas, focusing on early detection to limit the spread of the disease.
2. **Civil society engagement:** Involve civil society organizations in prevention and education efforts to enhance the reach and impact of public health campaigns, particularly in rural areas where traditional remedies remain prevalent.
3. **Geographical modulation:** Develop geographically tailored control strategies based on the specific bioclimatic zones of Morocco, targeting the vectors and reservoirs prevalent in each region
4. **Pharmaceutical advancements:** Promote the development of new pharmaceutical interventions, including vaccines and more effective treatments, to provide long‐term solutions for both cutaneous and VL.
5. **Pesticide strategy:** Establish a strategic plan for sustainable pesticide use to manage IR and ensure the long‐term effectiveness of vector control efforts.
6. **AI integration:** Leverage AI technologies to improve disease surveillance, enhance diagnostic accuracy, and optimize vector control strategies, making Morocco a leader in the use of cutting‐edge technology for managing NTDs.

By embracing these strategies, Morocco can make significant strides in controlling leishmaniases and protecting public health. Moving forward, policymakers should recognize the transformative potential of AI and prioritize its integration into disease management strategies. The adoption of AI can revolutionize Morocco′s approach to tackle leishmaniases, positioning the country as a regional leader in the use of advanced technologies for public health. Ultimately, this holistic and coordinated response will be essential for reducing the disease burden and improving health outcomes for affected populations across the diverse regions of Morocco.

## Funding

No funding was received for this manuscript.

## Conflicts of Interest

The authors declare no conflicts of interest.

## Data Availability

Data sharing is not applicable to this article as no datasets were generated or analyzed during the current study.
